# Current Methods in Synovial Fluid Microbiota Characterization: A Systematic Review

**DOI:** 10.3390/ijms26104690

**Published:** 2025-05-14

**Authors:** Elena Bardi, Daniele D’Arrigo, Chiara Pozzi, Andrea Gatti, Luca Bertolino, Alberto Favaro, Maria Rescigno, Tommaso Bonanzinga

**Affiliations:** 1 IRCCS Humanitas Research Hospital, Via Manzoni 56, Rozzano, 20089 Milan, Italy; elena.bardi@humanitas.it (E.B.); chiara.pozzi@humanitasresearch.it (C.P.); andrea.gatti@humanitasresearch.it (A.G.); alberto.favaro@humanitas.it (A.F.);; 2 Humanitas University, Via Rita Levi Montalcini 4, Pieve Emanuele, 20072 Milan, Italy; luca.bertolino@st.hunimed.eu; 3 Department of Biomedical Sciences, Humanitas University, Via Rita Levi Montalcini 4, Pieve Emanuele, 20072 Milan, Italy

**Keywords:** microbiota, microbiome, osteoarthritis, OA, synovial fluid, synovia, next generation sequencing, NGS

## Abstract

Evidence suggests that a cross-talk between the gut microbiota and joint health exists in a paradigm known as the gut–joint axis. Recent studies have also reported the presence of microorganisms potentially involved in the pathogenesis and progression of arthritis in synovial joints, previously believed to be sterile. This systematic review describes in detail the methodologies employed to characterize the microbiota in human synovial fluid (SF). A literature search was conducted in PubMed, Embase, and Web of Science up to 5 February 2025. Nine studies aimed to characterize the SF microbiome using next-generation sequencing or polymerase chain reaction. Eight studies detected bacterial DNA in SF. However, significant heterogeneity and incomplete reporting in methodologies, including sample collection and preparation, contamination management, DNA extraction and amplification, sequencing technology, targeted 16S rRNA or ITS regions, and bioinformatics processing, limit the comparability and significance of findings. Given the potential implications for understanding arthritis mechanisms and developing targeted treatments, a standardized methodological and reporting approach in SF microbiota characterization is needed to enhance the reproducibility and the relevance of results.

## 1. Introduction

Osteoarthritis (OA) is a degenerative disease affecting the cartilage, synovium, ligaments, and subchondral bone. As part of the disease process, synovial fluid (SF), which lubricates and nourishes the joint, is involved in OA pathogenesis and is used to identify biomarkers that are useful for diagnosis.

As estimated by the 2021 Global Burden of Diseases Study (GBD), OA is the most prevalent form of arthritis worldwide, affecting 7.6% of the population [1]. Including symptoms such as pain, stiffness, limited range of motion, and swelling, OA affects the mobility and the quality of life of patients [2]. Considering its high incidence and the severity of its consequences, OA represents one of the leading causes of disability among the elderly and a significant economic burden on healthcare systems [3,4]. Given the growing aging population and rising rate of obesity, the GBD forecasted an increase in OA prevalence by 48.6 to 95.1% between 2021 and 2050, making it an increasingly significant medical, social, and economic burden [1].

In an effort to avoid or at least postpone surgery, further in-depth analysis of pathogenesis is fundamental to find triggers for OA, implement strategies to prevent or delay disease progression, and improve treatment outcomes. OA is indeed recognized to involve not only mechanical processes but also local and systemic inflammatory responses [5,6], as well as metabolic factors [7,8]. In this context, with the advent of next-generation sequencing (NGS), researchers have shown increasing interest in the microbiota as a potential agent influencing inflammatory responses and the progression of OA. This cross-talk between the gut and joint microbiota is referred to as the gut–joint axis [9].

Dysbiosis of the gut microbiota is associated with increased levels of lipopolysaccharides (LPS) and pro-inflammatory factors [10], along with changes in the categories and components of short-chain fatty acids, which play a fundamental role in modulating gut permeability [11]. A weakening of the gut epithelial barrier allows inflammatory factors and bacterial components to leak into the bloodstream and trigger immune response and systemic inflammation, thus accelerating OA [11,12]. Furthermore, Huang et al. observed that increased levels of LPS in SF are positively associated with osteophyte severity, a reduced gap in the knee joint space, macrophage activation, and damage-associated molecular patterns [13].

Even though understanding the gut–joint axis brings insights into OA pathogenesis and potential alternative treatments, the gut microbiota might not be the only microbial player in joint health. In fact, different research groups detected the presence of a native microbiota inside the osteoarthritic joint, an environment previously thought to be sterile [14,15,16,17,18,19]. *Proteobacteria*, *Actinobacteria*, *Firmicutes*, *Fusobacteria*, and *Bacteroidetes* were the most common phyla identified. Moreover, the *Proteobacteria* phylum was more prevalent in OA knees compared to non-OA knees [20]. Despite the potential significance of these findings, trials investigating the presence of intra-articular microbiota exhibit great heterogeneity in terms of variation in sample sizes, sample collection methods, participant characteristics, possible contamination management, sequencing technique, and data analysis, limiting their impact and comparability [20,21,22].

A deeper understanding of the joint’s microbiota could shed further light on OA pathogenesis and positively affect its prevention, diagnosis, and management. With this end in mind, the purpose of this systematic review is to offer a comprehensive and detailed description of the methodologies employed to characterize the microbiota in native human SF, highlighting best practices to advance research in the field.

## 2. Materials and Methods

### 2.1. Database Search

This systematic review was conducted in accordance with the guidelines outlined by the Preferred Reporting Items for Systematic Reviews and Meta-Analysis (PRISMA), without a dedicated review protocol, and was not registered [23]. A scoping search was performed to identify the most appropriate keywords and determine the extent of the topic. Subsequently, a database search was conducted in PubMed, Embase, and Web of Science up to 5 February 2025, using the following query: (microbiota OR microbiome OR microflora) AND (synovial fluid OR synovia).

### 2.2. Study Selection Process

The inclusion criteria defined for conducting the screening process were as follows: (1) studies including characterization of the microbiota (in terms of bacteria species identification) (2) in the SF (3) of human joints (4) written in English. SF was chosen as the sample to investigate because it encompasses the entire joint environment and is commonly used in diagnostic procedures to identify biomarkers. The excluding criteria were as follows: (1) reviews and meta-analysis, case reports, opinions, conference abstracts, presentations, and editorials; (2) animal or in vitro assays; (3) studies having the main focus on infections; (4) studies targeting specific bacteria; (5) articles for which text was not available to the authors; and (6) articles written in a language other than English.

The database search outputs were imported into Rayyan (Cambridge, MA, USA) to facilitate duplicate removal and screening. After duplicate removal, two independent observers screened the titles and abstracts of the retrieved records and selected the relevant ones according to the inclusion and exclusion criteria listed above. The full text of the remaining articles was then screened following the same criteria. Finally, the reference lists of the selected articles were inspected to include as many studies as possible. Discrepancies between reviewers were resolved by discussion and consensus throughout the screening process. A senior investigator approved the results.

### 2.3. Data Extraction and Synthesis

Two reviewers examined the full text of the selected articles to collect the following pieces of information:

- sample collection, handling, and processing;
- DNA extraction method;
- library preparation (targeted regions, amplification, and contaminant control);
- sequencing technology;
- bioinformatics pipeline (quality filtering and denoising algorithms, clustering and classification, and quality control);
- downstream analyses (i.e., microbial diversity or others).

## 3. Results

### 3.1. Database Search and Study Selection

**Figure 1:**
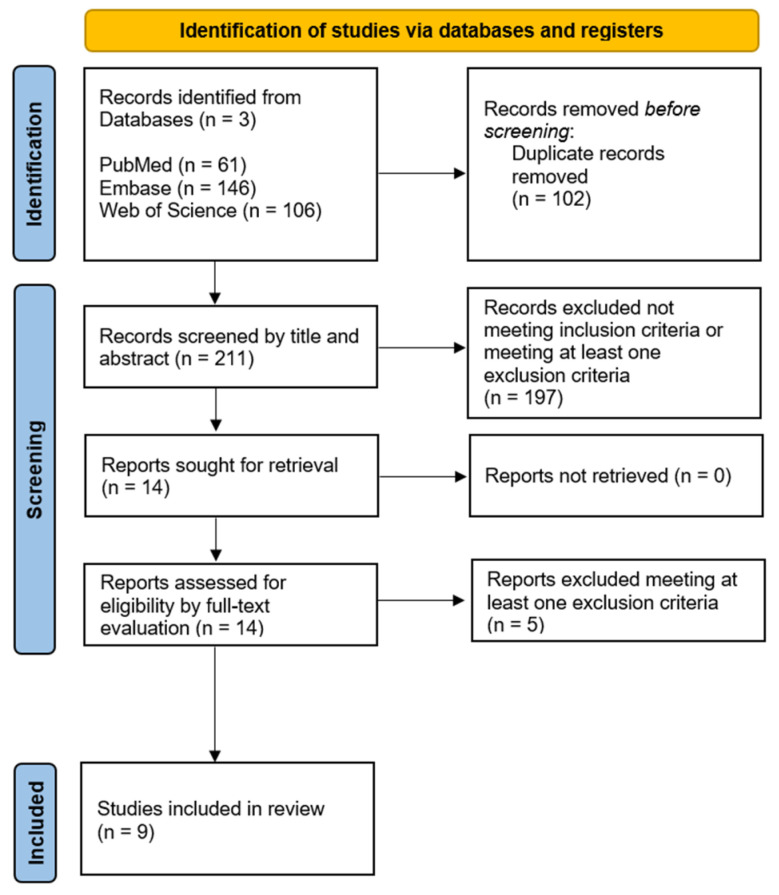
PRISMA 2020 flow diagram summarizing the study selection process [23].

The database search resulted in 313 records, of which 102 were duplicates and were removed. The titles and abstracts of the remaining 211 articles were screened, leading to the full-text inspection of 14 articles, of which 9 were ultimately included in the present review (Figure 1).

### 3.2. Data Extraction

Table 1 provides an overview of the studies included in the review, summarizing the study’s objective, population, investigation, and main results from microbiome analysis.

The participants enrolled were OA patients in six studies, rheumatoid arthritis (RA) patients in three, spondyloarthritis (SpA) patients in one, patients with prosthetic joint infection (PJI) in one, and patients with prosthesis aseptic failure in one. Healthy donors were included in four studies. Samples were collected before arthrotomy but after skin incision for OA, revision for PJI or aseptic failure, and during therapeutic aspiration for RA. Overall, microbiome sequencing of SF samples was performed on 743 participants. Of these, 293 (39.4%) were affected by OA, 397 (54.4%) by RA, and 3 (0.4%) by SpA, 12 (1.6%) were undergoing revision for PJI, 14 (1.9%) were undergoing revision for aseptic failure, and 24 (3.2%) were healthy. Of the nine studies included, eight detected microorganisms in SF, while one obtained negative results [27].

Methodological details for investigating the microbiota of knee intra-articular SF, including sample collection and processing, DNA extraction, library preparation, sequencing technology, the bioinformatics pipeline, and analyses conducted are summarized in Table 2.

All the studies aimed at characterizing the microbial community, with three also investigating the fungal community [16,18,26]. Of the nine studies, seven performed NGS [14,15,16,17,18,25,26], while two relied on either a broad-band or real-time polymerase chain reaction (PCR) alone [24,27]. Primers used for PCR amplification targeted the 16S rRNA hypervariable regions. One study alone specifically targeted *Firmicutes*, *Bacteroidetes*, *Proteobacteria (delta and gamma)*, and *Actinobacteria*, and the 16s rRNA region was used as denominator for the quantification of the phyla [24]. Among the studies targeting the hypervariable regions of the 16s rRNA gene, four targeted V1–V2, one targeted V2–V3 [15], and one targeted V4 [26]. Three studies did not specify the specific regions targeted [18,24,27], while two provided the exact primer sequences [14,24]. In all the three studies characterizing the fungal community, the primers used for PCR amplification targeted the ITS region [16,18,26].

SF was collected using aseptic technique from the knee in eight studies and from the hip in one study [17]. One remaining study provided a general description without specifying the joint from which the sample was collected or the procedure [27]. The amount of SF collected, which was reported by five of the nine studies considered, varied between 1 and 5 mL. The collected samples were stored at −80 °C in one study [26], or at −135 °C without heparin or hyaluronidase in two studies [14,25]. Only one reported the shipment at ambient temperature, as requested by the receiving lab facility [18].

All studies but one performed DNA extraction prior to PCR. Seven of them specified the kit used for extraction (all except one [18]), and three specified conducting mechanical lysis (or homogenization) before extraction [14,15,25]. One study skipped the extraction step to minimize laboratory-related contaminants [26].

Each study described PCR with a very different level of detail. Eight studies reported strategies for the management of contaminants, which mainly consisted in analyzing controls in parallel with SF samples to recognize contaminants introduced during sample collection, DNA extraction and PCR, and generic laboratory procedures.

Of the seven studies performing sequencing, three used Illumina MiSeq [15,16,26], two used Ilumina HiSeq [14,25], one used IonTorrent PGM4 [18], and one used both IonTorrent PGM4 and Illumina MiSeq [17].

The bioinformatics pipeline varied greatly in terms of both reporting and techniques between the seven studies employing NGS. QIIME was reported as the software package employed to carry out the processing and analysis of three of them [14,25,26]. The FLASH method was mentioned as the tool used to merge paired-end reads from sequencing by two [14,25]. Filtering was explicitly mentioned by four and included quality filtering and chimera removal, employing de novo chimera detection with UCHIME in three [14,16,25]. All studies clustered sequences into operational taxonomic units (OTUs), and three of them reported using an open-reference approach, which consists in a reference-based clustering against a database followed by a de novo approach [14,25,26]. The database against which OTUs clustering and/or taxonomic classification was conducted was specified in four studies and included GreeneGene, SILVA, NIH/Gen-bank, and ITS [14,18,25,26]. Two studies reported removal of commonly recognized contaminants OTUs, contaminants identified by control, OTUs considered as “no-hit”, and OTUs classified below a certain relative abundance [15,17]. Three studies also reported conducting a rarefaction procedure before diversity analysis [14,15,26].

Microbial diversity analysis included reporting the relative abundance for all the studies that successfully identified microorganisms. Three studies reported alpha-diversity calculated as the exponent of Shannon diversity (Hill1), or the Shannon index [14,17,24]. Among these, one also reported beta-diversity, evaluated as the Bray–Curtis dissimilarity [17]. Principal coordinates analysis (PCoA), either not specified or based on the Bray–Curtis dissimilarity matrix, or unweighted and weighted UniFrac distances, was instead conducted in three studies [14,17,26]. Among these, one also conducted prediction of functional composition with PICRUSt using KEGG pathways [14].

## 4. Discussion

The present review aimed to describe current methods of microbiome characterization for intra-articular SF as described in the literature. Overall, our review led to the selection of nine original research papers, with differing methodological approaches. Seven of these studies performed 16S rRNA amplicon sequencing on high-throughput NGS platforms IonTorrent and the more recent Illumina to characterize the entire bacterial and fungal microbiota. One study employed species-specific PCR to detect *Firmicutes*, *Bacteroidetes*, *Proteobacteria*, and *Actinobacteria*, which had previously been reported in intra-articular environments [24], and another employed broad-band PCR [27]. The choice of different methodological approaches was coherent with the specific purpose of the investigation, with studies implementing PCR in virtue of the method’s high specificity for detecting known sequences, especially in the presence of low abundance targets and NGS for analyzing large and complex unknown microbiomes in virtue of the enhanced sensitivity. Noteworthy is that none of the studies reviewed used shotgun metagenomics, which consists in sequencing the entire DNA avoiding primer-related bias altogether and features the advantages of a more comprehensive search and discrimination at the species level. However, this third-generation method is still not widely used or recommended for samples with a low bacterial load. The low microbial DNA content requires extremely deep sequencing to provide reliable results due to the high abundance of host genomic DNA. Additionally, from a computational perspective, this approach would demand significant processing effort and cost.

The comparison among the studies using NGS evidenced a preference for primers targeting the 16S rRNA gene hypervariable V1–V2 region [14,16,17,25] over V2–V3 [15] or V4 region [26]. Several authors suggest that the use of one subset over another can affect the accuracy and resolution of bacterial identification, resulting in under- or over-representation of specific taxa based on the regions chosen [28,29,30]. The choice of the hypervariable region also affects compatibility with reference databases, influencing the accuracy of taxonomic assignments [30]. One way to address the challenge of selecting the most suitable hypervariable region is to analyze the entire V1–V9 region simultaneously. This approach not only enhances the accuracy of taxonomic classification but also provides a more comprehensive representation of microbial diversity, reduces potential biases associated with single-region selection, and improves the resolution for detecting both abundant and rare taxa. Additionally, using the full V1–V9 region can enhance comparability across studies and better capture phylogenetic relationships.

While the description of platforms and targeted hypervariable regions was equally reported across most studies, the description of technical aspects relevant to the replication and interpretation of the study findings was more heterogeneous in terms of the level of detail, likely reflecting the novelty of this field of investigation and the lack of specific reporting guidelines for these types of studies. In recent years, several resources, including the International Human Microbiome Standards (IHMS) and the Strengthening The Organization and Reporting of Microbiome Studies (STORMS) guidelines and checklist, have provided guidance on key items to be reported in microbiome studies, the variability of which can potentially introduce relevant bias to NGS data [28,31,32,33]. According to these, relevant details that should be reported include information on patient population and environmental exposure/geographical origin, sample collection and processing, DNA extraction methods and kits, contamination mitigation and quality control, data cleaning, human DNA depletion, batch effects, and minimum input for detection and accessory analyses [28,33].

With specific reference to the initial phase of sample collection, handling, and processing, best practices are that the working conditions, operators, and equipment remain consistent throughout, since any source of variability could affect the NGS data analysis [28,33]. In our review, all studies had contamination prevention and control protocols in place. Most studies mentioned national or facility-specific protocols providing details on disinfection procedures, aseptic techniques, or handling in sterile environments. Torchia et al. carried out environmental sampling by collecting sterile water samples from a container in the operating room at different time points to detect environmental contaminants upon sample collection [18]. Two studies placed an open PBS tube in the operating room during the procedure [14,25]. Moreover, all studies mentioned the run of negative controls throughout the sequencing process, with few studies providing details on the rate of running negative controls and further processing for DNA quantification in negative samples [16,24,26].

In terms of the amount of sample collected, this consisted of at least 1 mL across all studies. Indeed, it is acknowledged that inadequate amounts of samples increase the risk of false negatives and false positives as well as fail to capture microbial diversity. Moreover, it may lead to under-sequencing, overfitting in statistical models, and the missed detection of rare variants that are not adequately represented. In general, the choice of the amount of sample is based on the purpose of the study and the depth of coverage required, as well as cost restraints.

Other aspects that need to be considered include the technological upgrade over time of the NGS platforms used. Although the outputs of IonTorrent and the more recent Illumina have been acknowledged as comparable [17], so far, no studies have compared the sensitivity and specificity of these platforms in the detection of joint microbiome. The issue of technical changes over time is also discussed by Borsinger et al., who reported discrepancies in microbial detection via NGS compared to their previous work [16,18]. The authors highlighted that the manufacturer had utilized a different extraction method in the two studies, making direct comparison of the incidence of NGS positivity challenging.

Finally, bioinformatics analysis can introduce a considerable degree of bias during several steps. In the first step, low-quality sequences are filtered out, the remaining sequences are joined, and chimeras are identified and removed. This step was demonstrated to affect the detection of low-abundance organisms [33]. Even though such bias might not be particularly relevant for some scenarios, it might be problematic when SF is concerned, since the human joints were previously believed to be sterile and culture approaches usually show negative results, possibly due to a low abundance. A trade-off between the quantity of included low-quality reads and sensitivity for detecting low-abundance organisms should be sought [30].

Sequences can then be either clustered into OTUs or corrected by means of denoising algorithms and then assigned to amplicon sequence variants (ASVs). All the studies included in the review mentioned OTU clustering, which is more computationally efficient with respect to ASV identification, simplifies the analysis when fine resolution is not needed, and facilitates comparison with previous work. However, OTU clustering has been proven to overestimate microbial diversity and richness [34]. On the other hand, ASVs’ higher resolution allows distinguishing between similar species, and the algorithms employed guarantee a higher reproducibility and comparability of results. The use of rarefaction prior to diversity metric computations could provide more robust/concordant results between ASV- and OTU-based approaches [34]. However, only three of the seven studies performing NGS reported applying rarefaction analysis.

The quality and completeness of the reference database against which OTUs clustering and taxonomic assignment are made (SILVA, GreenGenes, NIH/Gen-bank, or ITS) is of paramount importance in determining the accuracy of microbiota characterization. In particular, the SILVA and RDP databases were found to have superior performances with respect to GRD, LTP, and GreenGenes at the genus level [30]. Despite this, not all studies specified the database against which this process was carried out, nor the alignment algorithm or the identity threshold employed. Given the importance of the choice of database for the accuracy of the results, the database used for taxonomic classification and the algorithm employed for alignment should be clearly reported.

Once the bioinformatics processing is complete, a good microbiota analysis generally starts by calculating relative abundances. This is followed by alpha- and beta-diversity analyses, which describe the community within a sample or between different sample groups. Alpha-diversity was computed in three of the studies included [14,17,24], while beta-diversity was analyzed in two of these studies plus one [14,17,26]. Normally, beta-diversity includes PCoA to visualize and interpret the differences in community composition between samples by reducing high-dimensional data projecting it into a lower-dimensional space. In this case, bias can be introduced by the choice of the diversity metric employed, since different metrics express slightly different diversity characteristics and can affect the power of the analysis [35].

Two of the studies conducting microbial diversity analysis employed the Shannon index as the metric to express alpha-diversity, while one employed Hill1, computed as the exponent of the Shannon diversity [17]. The Shannon index is a measure of entropy and considers both the presence and the abundance of each taxa, combining richness and evenness. Hill1 instead converts the Shannon index into a number of species, making it more intuitive to interpret. Concerning beta-diversity, one study employed UniFrac distances [14], while the other two employed Bray–Curtis dissimilarity [17,26]. The first takes into account the phylogenetic tree, representing evolutionary relationships, and the distances between community members. Unweighted UniFrac considers only the presence or absence of taxa, while weighted UniFrac also considers differences in abundance. Bray–Curtis dissimilarity, on the other hand, measures the compositional dissimilarity between two samples based on species count and does not include phylogenetic relationships. Shannon’s and Bray–Curtis metrics are reported to be the most used in the literature, possibly because of their higher sensitivity to observe differences between groups that require smaller sample sizes [35].

Finally, statistical analysis such as differential abundance can be conducted to obtain information not only about communities but also about individual taxa that are significantly different between groups. Functional prediction can be additional, but not essential [14].

## 5. Conclusions

The presence of a microbial community in native human joints is a recent concept challenging the dogma of the sterility of the synovial environment. Further research is necessary to provide evidence to sustain this concept and deepen the understanding of potential correlations with joint health, aiming to identify specific treatments targeting the microbiota. Currently, microbiota analysis in synovial fluid presents significant heterogeneity in both the methodologies used and the reporting of the results. This heterogeneity spans from sample collection procedures to DNA extraction and amplification, sequencing, bioinformatics pipeline, and microbial diversity analysis, introducing considerable bias. Such bias not only limits the impact of the results but also prevents comparisons across studies. Adhering to the standard operating procedures identified by the IHMS and reporting checklists like STORMS are crucial to mitigate bias and to foster meaningful progress in this field.

## Figures and Tables

**Table 1:** Summary of the studies investigating the microbiota of synovial fluid.

Authors, Year	Aim	Population	Analyses	Results
Elsawy, 2024 [24]	Assess the relation between microbiome and LPS in the blood and SF with FCT knee OA patients	40 patients with knee OA (F: 72.5%)—Egypt	• PCR for microbiomes and ELISA for LPS in serum and SF • US for FCT	• 100% OA patients had bacterial DNA in SF • *Firmicutes* was the most abundant (63.59%), followed by *Actinobacteria* (24.14%), *Proteobacteria* (11.51%), and *Bacteroidetes.*
Fernandez-Rodríguez, 2023 [15]	Investigate presence of the microbiome in human knee and compare the profile in different knee conditions	15 healthy volunteers, 14 patients with OA undergoing TKA, 12 septic revision surgery, 10 contralateral knee non-OA, 14 aseptic revision surgery (F: 50.9%)—United States	Culture and NGS analysis of SF	• 13.8% positive SF cultures • Highest number of species in native OA knees • *Cutibacterium*, *Staphylococcus*, and *Paracoccus* dominant in non-OA knees • *Proteobacteria* dominant in OA knees • Similar trend in composition for contralateral and aseptic revision knees • NGS of PJI SF confirmed culture results
Goswami, 2023 [17]	• Explore the microbial composition within joints of OA patients during joint arthroplasty • Quantify impact of factors associated with microbiome activity and their source	117 patients with hip or knee OA undergoing arthroplasty from 13 institutions—United States	NGS of SF, ST, and swab specimens	• 87% of SF samples positive to NGS, only 38% ≥ 800 reads • *Escherichia*, *Cutibacterium*, *Staphylococcus*, *Acinetobacter*, and *Pseudomonas* were the most abundant genera • Hospital explained a portion (18.5%) of the variance in composition • Corticosteroid injection associated with high abundance of several lineages
Borsinger, 2024 [16]	Evaluate the presence of microorganisms in bilateral native knees with OA	40 patients undergoing primary UKA (n = 30) or bilateral (n = 10) TKA (F: 54%)—United States	• NGS of SF, ST and swabs • SF biomarkers: CRP, WBC count, and %PMNs.	• Positive NGS in 3 of 80 samples (3.8%), two below threshold • Two multiple microorganisms identified (1 knee with 4 microorganisms; 1 knee with 2 microorganisms) • *Cutibacterium acnes* was the most common (2 out of 3 samples) species • No association between patient characteristic and positive NGS
Cheng, 2022 [25]	• Investigatestage-specific roles of microbial dysbiosis and metabolic disorders in RA • Comparison healthy vs. OA vs. RA	76 with RA (stages I–IV), 19 with OA and 27 healthy for fecal-plasma study, and 271 with RA (stages I–IV) for knee SF study (F: 77.9%)—China	• Stage-based profiles of faecal metagenome and plasma metabolome • SF NGS analysis, bacterial isolation and scanning electron microscopy	• Not enough bacteria DNA in stages I-III • Most of the microbes in SF were *Proteobacteria* and *Firmicutes*
Torchia, 2020 [18]	Characterize the native knee microorganism profile in patients undergoing TKA	40 patients with OA undergoing TKA (F: 52.5%)—Unites States	NGS for SF bacterial detection	• 30% of TKA patients with organisms identified by NGS • An average of 4.6 organisms found among patients with positive NGS results, and 48 unique organisms identified by NGS from all samples
Hammad, 2019 [26]	• Investigate SF for presence of bacterial and fungal DNA • Investigate association between bacterial and fungi community composition to synovial inflammation markers	16 patients with RA and 9 healthy volunteers (F: 48%)—United Kingdom	• NGS for SF bacterial and fungal detection • ELISA for inflammatory markers levels (IL-6, IL-17A, IL-22, and IL-23)	• Bacterial DNA detected in the SF of 87.5% patients with RA, and in 100% healthy controls • Fungal DNA detected in 75% RA samples, and 89% healthy controls • SF predominated by *Proteobacteria* (Control = 83.5%, RA = 79.3%) and *Firmicutes* (Control = 16.1%, RA = 20.3%) and *Actinobacteria* (Control = 0.2%, RA = 0.3%) and *Bacteroidetes* (Control = 0.1%, RA = 0.1%) • SF predominated by members of the *Basidiomycota* (Control = 53.9%, RA = 46.9%) and *Ascomycota* (Control = 35.1%, RA = 50.8%)
Cristea, 2019 [27]	Identify which parameters best explain the correlation between SpA activity/severity andmicrobiological results/immune status against intestinal and/or urogenital pathogens.	27 patients with SpA and 26 healthy volunteers (F: 32.1%)—Romania	• Microbiological investigation (culture, nucleic acid-based assays) of stool, urine, SF and serum. • Screening of anti-thyroid antibodies and peroxidase • Detection of HLA-B27 by PCR and the determination of CRP biomarker	• SF from 3 SpA patients negative on culture and PCR analysis
Zhao, 2018 [14]	Characterize potential SF bacterial nucleic acids	125 patients with RA (5 ST and 110 SF samples), and 58 with OA (16 ST and 42 SF samples) (F: 83.1%)—China	NGS for bacterial detection	• Abundant diversity of bacterial DNA, including *Porphyromonas* and *Bacteroides* in 100% samples • *Veillonella dispar*, *Haemophilus parainfluenzae*, *Prevotella copri* and *Treponema amylovorum* more abundant in SF of RA • *Bacteroides caccae* more abundant in SF of OA

LPS: lipopolysaccharides, SF: synovial fluid, FCT: femoral cartilage thickness, OA: osteoarthritis, PCR: polymerase chain reaction, US: ultrasound, TKA: total knee arthroplasty, NGS: next generation sequencing, PJI: periprosthetic joint infection, ST: synovial tissue, UKA: unilateral knee arthroplasty, CRP: C-reactive protein, WBC: white blood cells, PMNs: polymorphonuclear leukocytes, RA: rheumatoid arthritis, SpA: spondyloarthritis, F: female.

**Table 2:** Summary of methodological approaches in microbiota fluid characterization.

Author, Year	Sample Collection	DNA Extraction	Library Preparation		Sequencing	Bioinformatics Pipeline		Downstream Analyses
	Size, collection, sterility	Method—Kit	Targeted regions and amplification	Contaminants	Platform	Filtering/Denoising	Clustering and classification	
NGS								
Fernàndez-Rodrìguez, 2023 [15]	• knee • 3 mL min • aseptic technique • in the operating room • before arthrotomy/at the start of the elective procedure	• mechanical lysis with Qiagen TissueLyser • extraction with KingFisher Flex Purification System	• 16S rRNA V2-V3 •PCR on LightCycler 480 II (Roche Life Science)	negative extraction controls included	Illumina MiSeq	N/A	• clustering not directly reported • removal of commonly recognized contaminants, ‘No hit’ OTUs, OTUs below 2% of relative abundance	• rarefaction • relative abundance
Goswami, 2023 [17]	• hip and knee • 2 mL • aseptic technique • in the operating room • before arthrotomy • using 18-gauge needle	not directly reported but similar to methods described in previous studies	• 16S rRNA V1-V2 • PCR not directly reported but similar to methods described in previous studies	negative laboratory procedures controls included	IonTorrent PGM4 and Illumina Miseq26	not directly reported but similar to processing described in previous studies	• clustering not directly reported but similar to processing described in previous studies • removal of contaminants identified by controls with no PCR template, OTUs detected in <1% of samples	• relative abundance • α-diversity: Hill1 (exponent of the Shannon diversity) • β-diversity: Bray–Curtis dissimilarity PCoA
Borsinger, 2024 [16]	• knee • 1 cm 3 min • aseptic technique • in the operating room • before arthrotomy after skin incision (for the operative knee) • aseptic aspiration (for the non-operative knee) • transport in sterile containers	extraction with Zymo Research kit	• 16S rRNA V1-V2 and ITS • PCR on Applied Biosystems • products combined based on qualitative band strength • size selection with Agencourt AMPure XP beads and Qiagen Minelute Kit • library quantified with Qubit 4.0 fluorometer	3 negative extraction controls run every 92 samples	Ilumina MiSeq	• de novo chimera removal in UCHIME • removal of any read below the quality score, quality metric, or length • removal of any sequence that did not contain a valid barcode (demultiplexing with internally developed algorithm)	• OTU global alignment using USEARCH against a database of high-quality sequences • species below 2% reported as number of reads	• percent dominance
Cheng, 2022 [25]	• knee • 5 mL • aseptic technique in sterile atmosphere • during therapeutic aspiration • placed in sterile tubes on ice and homogenized within 5 min of collection, immediately frozen and kept w/o heparin or hyaluronidase at −135 °C	extraction with TIANGEN bacterial DNA kit	• 16S rRNA V1-V2 • PCR not directly reported	environmental control (PBS tube left open during the procedure) run in parallel	Illumina HiSeq 2500	• reads trimmed for adapter and primer sequences, splitted according to the barcodes in QIIME • Reads with average quality less than 25 were removed • sequences joined with FLASH method • de novo chimera removal with USEARCH	• open reference OTU picking protocol QIIME at 97% sequence similarity • RDP classifier vs. GreenGene database with 0.8 confidence.	• relative abundance
Torchia, 2020 [18]	• knee • 1 cm3 • in the operating room • before arthrotomy after skin incision • tube placed in sterile container • shipped at ambient temperature	N/A	• 16S rRNA and ITS • emulsion PCR	environmental control (four operative field sterile water collected at different time points) run in parallel	IonTorrent PGM4	N/A	cross-reference of the sequences against the NIH/Gen-bank database with 90% agreement	• relative quantity (DNA load)
Hammad, 2019 [26]	• knee • aseptic technique • aspiration (either therapeutic or for donation) • transferred to sterile micro-centrifuge tube at −80 °C	no extraction to avoid contaminants	• 16S rRNA V4 and ITS2 • direct PCR: two rounds to add sequencing adapters • purification with AMPure XP magnetic beads • DNA quantification using Qubit 3.0 hsDNA kit	one negative reaction control (UV-irradiated nuclease-free water) run in parallel during both reactions	Ilumina MiSeq	Nephele 16S/ITS paired-end QIIME pipeline	open reference OTU clustering against SILVA database for bacteria and ITS for fungi at 99% sequence identity in QIIME	• rarefaction • relative abundance • PCoA based on Bray–Curtis distance matrix
Zhao, 2018 [14]	• knee • aseptic technique in sterile environment • during therapeutic aspiration • placed in sterile tubes on ice and homogenized within five minutes of collection, immediately frozen and kept w/o heparin or hyaluronidase at –135 °C	• extraction with DNeasy Blood and Tissue Kit • DNA quantitation with Nanodrop 2000	• 16S rRNA V1-V2 • PCR • amplicons purified using QIAquick PCR Purification Kit	sample collection controls, reaction mixture controls and environmental control (PBS tube left open during the procedure) run in parallel	Illumina HiSeq	• sequences joined and quality filtered with FLASH method • de novo chimera removal with USEARCH	• first OTU clustering against 2013 GreenGenes database at 97% similarity • second de novo OTU clustering at 97% similarity with UCLUST • QIIME RDP classifier against the GreenGenes data set • UPGMA hierarchical clustering (average linkage) on the distance matrix of OTU abundance • Newick formatted tree with the QIIME package	• rarefaction• relative abundance • α-diversity: Shannon index using OTU tables rarefied to the lowest number of reads obtained for any of the samples analyzed. • PCoA based on unweighted and weighted UniFrac distances using evenly sampled OTU abundances • Prediction of functional composition with PICRUSt using KEGG pathways
Real-time PCR								
Elsawy, 2024 [24]	• knee • aspiration in the operating room • aseptic technique	extraction with QIAamp DNA Mini kit	• 16S rRNA (as denominator) • Firmicutes • Bacteroidetes • Proteobacteria (delta and gamma) • Actinobacteria • Rotor Gene Q real-time PCR machine	negative extraction and reaction controls (sterile distilled water) included in each PCR reaction	N/A	N/A	N/A	• relative abundance • α-diversity: Shannon index
Broad range PCR								
Cristea, 2019 [27]	N/A	extraction with PureLink Genome DNA Mini kit	• 16S rRNA • Broad range PCR	N/A	N/A	N/A	N/A	N/A because there were no samples positive to PCR

TKA: total knee arthroplasty, NGS: next-generation sequencing, OTU: operational taxonomic unit, PCR: polymerase chain reaction.

## Data Availability

The original contributions presented in this study are included in the article.

## Abbreviations

The following abbreviations are used in this manuscript:

ASV	Amplicon sequence variant
GBD	Global Burden of Disease
IHMS	International Human Microbiome Standards
LPS	Lipopolysaccharides
NGS	Next-generation sequencing
OA	Osteoarthritis
OTU	Operational taxonomic unit
PCoA	Principal coordinate analysis
PCR	Polymerase chain reaction
PJI	Periprosthetic joint infection
PRISMA	Preferred Reporting Items for Systematic Reviews and Meta-analyses
RA	Rheumatoid arthritis
SF	Synovial fluid
SpA	Spondyloarthritis
STORMS	Strengthening The Organization and Reporting of Microbiome Studies
